# Efficacy and safety of buparlisib, a PI3K inhibitor, in patients with malignancies harboring a PI3K pathway activation: a phase 2, open-label, single-arm study

**DOI:** 10.18632/oncotarget.27251

**Published:** 2019-11-05

**Authors:** Sarina A. Piha-Paul, Matthew H. Taylor, Daniel Spitz, Lee Schwartzberg, J. Thaddeus Beck, Todd M. Bauer, Funda Meric-Bernstam, Das Purkayastha, Linda Karpiak, Sebastian Szpakowski, Fadi Braiteh

**Affiliations:** ^1^ Department of Investigational Cancer Therapeutics, The University of Texas MD Anderson Cancer Center, Houston, TX, USA; ^2^ Division of Hematology & Medical Oncology, Oregon Health and Science University, Portland, OR, USA; ^3^ Department of Hematology & Oncology, Florida Cancer Specialists & Research Institute, West Palm Beach, FL, USA; ^4^ Division of Hematology & Oncology, The West Clinic, Memphis, TN, USA; ^5^ Department of Oncology, Highlands Oncology Group, Fayetteville, AR, USA; ^6^ Department of Drug Development, Sarah Cannon Research Institute, Tennessee Oncology, PLLC, Nashville, TN, USA; ^7^ Novartis Pharmaceuticals Corporation, East Hanover, NJ, USA; ^8^ Novartis Institutes for Biomedical Research, Cambridge, MA, USA; ^9^ Department of Medical Oncology, US Oncology Research and Comprehensive Cancer Centers of Nevada, Las Vegas, NV, USA

**Keywords:** phosphatidylinositol 3-kinase pathway, buparlisib, advanced malignancies, molecular selection, tissue agnostic

## Abstract

**Background:** Phosphatidylinositol 3-kinase (PI3K) pathway activation plays a key role in tumorigenesis and has been associated with poor prognosis and resistance to multiple therapies in various cancers.

**Results:** There were 146 patients enrolled; common tumor types were colorectal, sarcoma, and ovarian. Tumors had PI3K pathway alterations and a median of four mutations with tissue-specific patterns of mutation burden (lowest: sarcoma [2.5]; highest: esophagus, germ cell tumor, skin non-melanoma, vaginal [7]). The number of prior therapies did not correlate with the number of genetic alterations (Pearson *r* = –0.037). The clinical benefit rate was 15.1% (*n* = 22). An additional patient had an unconfirmed complete response. The most common adverse events were fatigue, nausea, hyperglycemia, decreased appetite, and diarrhea.

**Patient and Methods:** In this phase 2, open-label, single-arm study, patients with solid or hematologic malignancies with PI3K pathway activation and progression on or after standard treatment received buparlisib (100 mg once daily). The primary endpoint was clinical benefit rate per local investigator assessment (response or stable disease at ≥16 weeks).

**Conclusions:** Buparlisib was well tolerated, however efficacy was limited despite selection of PI3K pathway aberrations. Future studies may provide insight into buparlisib efficacy by refining the molecular selection of different tumor types.

## INTRODUCTION

Phosphatidylinositol 3-kinase (PI3K) signaling regulates diverse cellular functions including cell proliferation, survival, translational regulation of protein synthesis, glucose metabolism, cell migration, and angiogenesis [1, 2]. Constitutive activation of PI3K signaling is a critical step in mediating tumorigenesis of many tumor types and can be linked to resistance to a variety of therapeutic interventions, including chemotherapy, hormonal therapy, and anti-human epidermal growth factor receptor 2 therapies [3]. Furthermore, preliminary data suggest that activation of the PI3K pathway may be a predictor of poor prognostic outcomes in many cancers [4].

Molecular changes leading to constitutive activation of the PI3K pathway are diverse and include, but are not limited to, gain-of-function mutations of PI3K subunits (*PIK3CA* gene encoding the PI3K catalytic subunit p110α; genes encoding the p85 regulatory subunit including *PIK3R1*) or oncogenes encoding positive regulators of PI3K (e. g. *HER2*, *EGFR*, *RAS*, Src-family proteins) [1, 5]; loss-of-function mutations of *PIK3R1* [6]; loss-of-function mutations or epigenetic alterations affecting negative regulators of PI3K signaling (e. g. loss of *PTEN* expression or function) [7, 8]; and amplification of the *PIK3CA* gene [5]. Based on these observations, the PI3K pathway has been a critical therapeutic target for the treatment of patients with advanced solid tumors [4, 9].

Buparlisib is a potent and highly specific oral pan-class I PI3K inhibitor that targets all four isoforms of class I PI3K (α, β, γ, δ), including the most common somatic *PIK3CA* mutants [10]. However, *in vitro* studies have shown that it does not significantly inhibit *mTOR* or Vps34 [10]. Consistent with its mechanism of action, buparlisib reduced cellular levels of phosphorylated protein kinase B and its downstream effectors both *in vitro* and *in vivo* [10]. Buparlisib has shown preliminary activity in preclinical models of solid tumors [11], and based on this, buparlisib has been evaluated in several clinical studies.

In this study (NCT01833169), buparlisib was investigated as part of the Novartis Signature Program, a series of eight signal-seeking phase 2 basket trials in patients with solid or hematologic tumors and an actionable mutation [12]. The Novartis Signature Program matched genetic alterations to targeted therapies in a tissue-agnostic fashion, with no pre-identified clinical trial sites. Each trial investigated a single agent and enrollment was based on histology-agnostic and mutation-specific criteria. In this trial, patients were pre-identified as having PI3K pathway-activated solid tumors and/or hematologic malignancies and subsequently matched to buparlisib treatment. The goal of this study was to determine whether treatment with buparlisib monotherapy demonstrated sufficient efficacy in PI3K pathway-activated tumors to warrant further study.

## RESULTS

### Patients

Between May 31, 2013 and September 26, 2016, 146 patients were enrolled and received at least one dose of buparlisib across 69 sites, including community-research networks (30 sites [44%]; 67 patients), independent community sites (27 sites [39%]; 30 patients), and academic sites (12 sites [17%]; 49 patients).

Patient baseline characteristics are presented in Table 1. The median age was 60 years (range, 22–86), 34.9% were aged 65 or older. Most patients (99.3%) had an Eastern Cooperative Oncology Group performance status of 0 or 1. Enrolled patients were heavily pretreated, with 52.7% having received at least three prior lines of therapy; the median number of prior therapies was three (range, 1–13). Overall, 140 patients had solid tumors and six patients had a tumor with unknown primary site. The most frequent tumor types were colorectal (12.3%), sarcoma (9.6%), and ovarian (8.2%). Among the 146 patients, 72 (49.3%) tumor samples were from primary lesions and the remaining 74 (50.7%) were from metastatic lesions.

**Table 1:** Patient baseline characteristics

Baseline characteristic	Patients (*N* = 146)
Median age (range), years	60 (22–86)
Age group (years), *n* (%)	
<65	95 (65.1)
≥65	51 (34.9)
Sex, *n* (%)	
Female	85 (58.2)
Male	61 (41.8)
ECOG, *n* (%)	
0	54 (37.0)
1	91 (62.3)
2	1 (0.7)
Tumor type,^a^ *n* (%)	
Colorectal	18 (12.3)
Sarcoma	14 (9.6)
Ovarian	12 (8.2)
Cervix	11 (7.5)
Head and neck squamous cell carcinoma	11 (7.5)
Anal	10 (6.8)
Prior therapies, *n* (%)	
Median (range)	3.0 (1–13)
1	29 (19.9)
2	40 (27.4)
3	21 (14.4)
4	19 (13.0)
≥5	37 (25.3)
Genetic analysis based on central assessment,^b^ *n* (%)	
*PIK3CA* mutation	50 (32.4)
*PTEN* mutation or loss (non-immunohistochemistry)	29 (19.9)
PTEN loss by immunohistochemistry^c^	23 (15.8)
*PIK3CA* amplification	10 (6.8)
*PIK3R1* mutation	6 (4.1)

^a^Other tumors in <5% of patients were gall bladder, gastroesophageal junction (*n* = 6 each), bladder (*n* = 5), liver, gall bladder ducts, neuroendocrine, skin non-melanoma, small intestine, thyroid, vaginal (*n* = 4 each), esophagus, germ cell tumor, pancreas (*n* = 3 each), melanoma, salivary gland, appendix (*n* = 2 each), and unknown primary (*n* = 6). Other histologies (*n* = 4) included only one patient each.

^b^An individual patient could be counted in multiple categories.

^c^PTEN loss by immunohistochemistry is based on local assessment.

Abbreviation: ECOG, Eastern Cooperative Oncology Group.

The most common genetic aberrations were *PIK3CA* gene mutations (34.2%; *n* = 50) or *PTEN* gene aberrations (19.9%; *n* = 29) (Table 1). *PIK3CA* amplifications and *PIK3R1* mutations were identified in 6.8% (*n* = 10) and 4.1% (*n* = 6) of patients, respectively. The median time from tumor biopsy used for sequencing (performed at disease diagnosis or later) to first dose of study treatment was 13.6 months (range, <1–124). The median time from initial diagnosis to first dose of study treatment was 26.5 months (range, 3–276).

The median duration of treatment was 1.8 months (range, 0–21.2). Overall, 39.0% of patients had a dose interruption or reduction. The most frequent reasons for any dose delays or reductions were adverse events (30.1%), per protocol (24.0%), and laboratory test abnormalities (11.0%).

All patients have discontinued study treatment. The primary reasons for discontinuation were disease progression (67.8%) or adverse events (22.6%). The most common adverse events (≥three patients) which led to study drug discontinuation were depression (2.7%), increased lipase (2.1%), hyperglycemia (2.1%), and anxiety (2.1%).

### Efficacy

Of 146 patients, 117 (80%) were eligible for efficacy assessments per Response Evaluation Criteria In Solid Tumors (RECIST) 1.1 (measured from time of first dose to the date of first documented disease progression, relapse, or death). The clinical benefit rate was 15.1% (*n* = 22; primary endpoint; one partial response and 21 stable disease; Table 2). One patient with vaginal cancer achieved complete response at the cycle 3, day 1 visit but discontinued due to adverse events before week 16 and was therefore not evaluable for clinical benefit; this patient had received one prior line of treatment. The overall response rate was 1.4% (one complete response and one partial response; Table 2). The duration of response was 113 days for the patient who achieved a confirmed partial response and 29 days for the patient who achieved an unconfirmed complete response, although a lack of available follow-up evaluations prevents determination of the complete response duration.

**Table 2:** Summary of overall response rate and clinical benefit rate

Tumor response, *n* (%)	Patients (*N* = 146)
Complete response	1 (0.7)^a^
Partial response	1 (0.7)
Stable disease	21 (14.4)
Progressive disease	94 (64.4)
Non-evaluable	29 (19.9)
Overall response rate^b^ *n* (%), 95% confidence interval^c^	2 (1.4) [0.2–4.9]
Clinical benefit rate^d^ *n* (%), 95% confidence interval^c^	22 (15.1) [9.7–21.9]

^a^Patient achieved a complete response but was discontinued due to an adverse event before week 16 and was therefore not evaluable for clinical benefit.

^b^Overall response rate = complete response + partial response.

^c^Exact binomial confidence interval using Clopper–Pearson method.

^d^Clinical benefit rate = complete response + partial response + stable disease ≥16 weeks.

**Figure 1:**
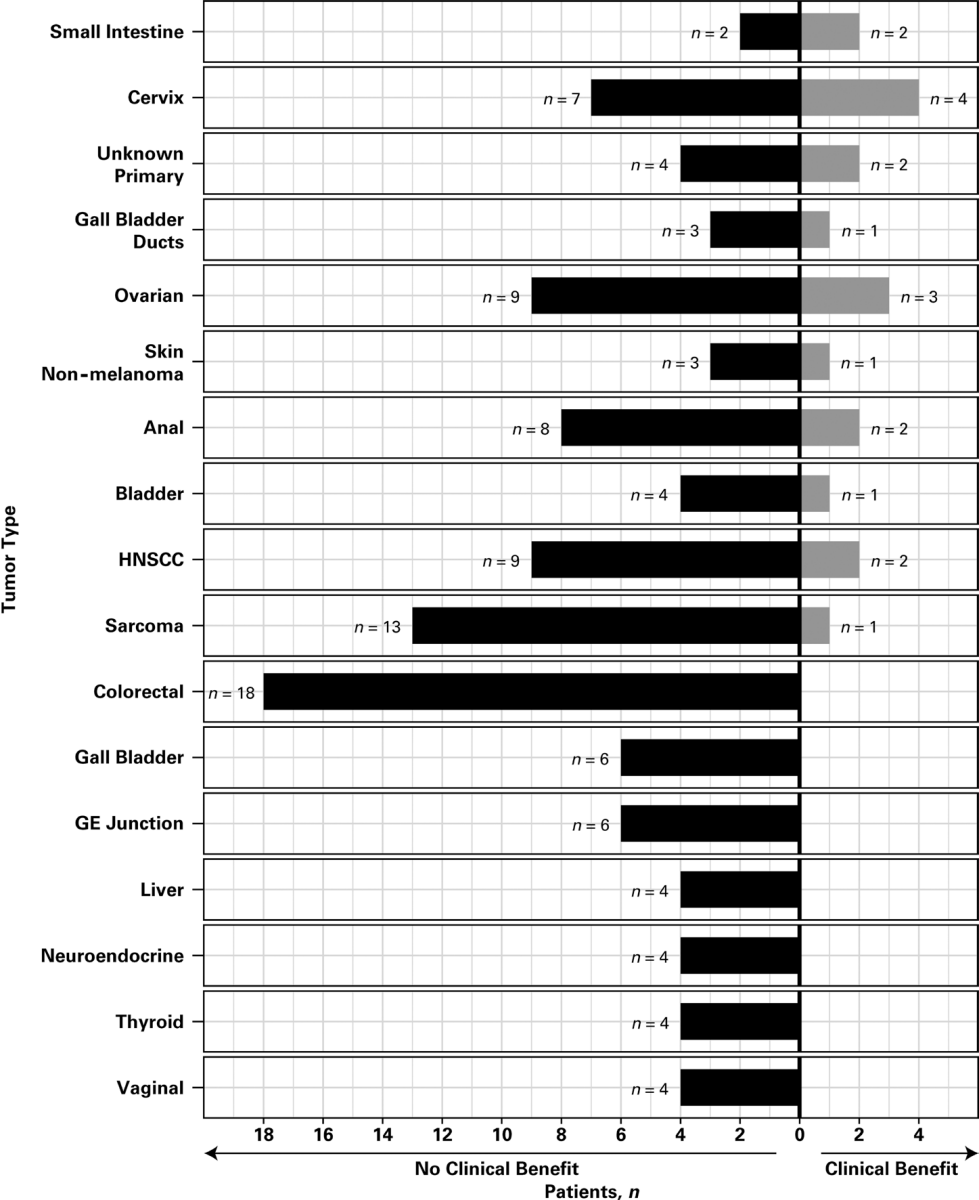
Clinical benefit according to tumor type. Only tumor cohorts with at least four patients are included. Abbreviations: GE, gastroesophageal; HNSCC, head and neck squamous cell carcinoma.

Among the 17 tumor cohorts with at least four patients, clinical benefit was observed for at least one patient per cohort in most tumor types, except colorectal, gall bladder, gastroesophageal junction, liver, neuroendocrine, thyroid, and vaginal (Figure 1). No tumor cohort was considered successful according to the Bayesian analysis. The clinical benefit rate in the skin non-melanoma cohort exceeded the assumed historical control rate with one of four patients experiencing clinical benefit. No other tumor cohort reached a clinical benefit rate that exceeded the assumed historical control rate.

**Figure 2:**
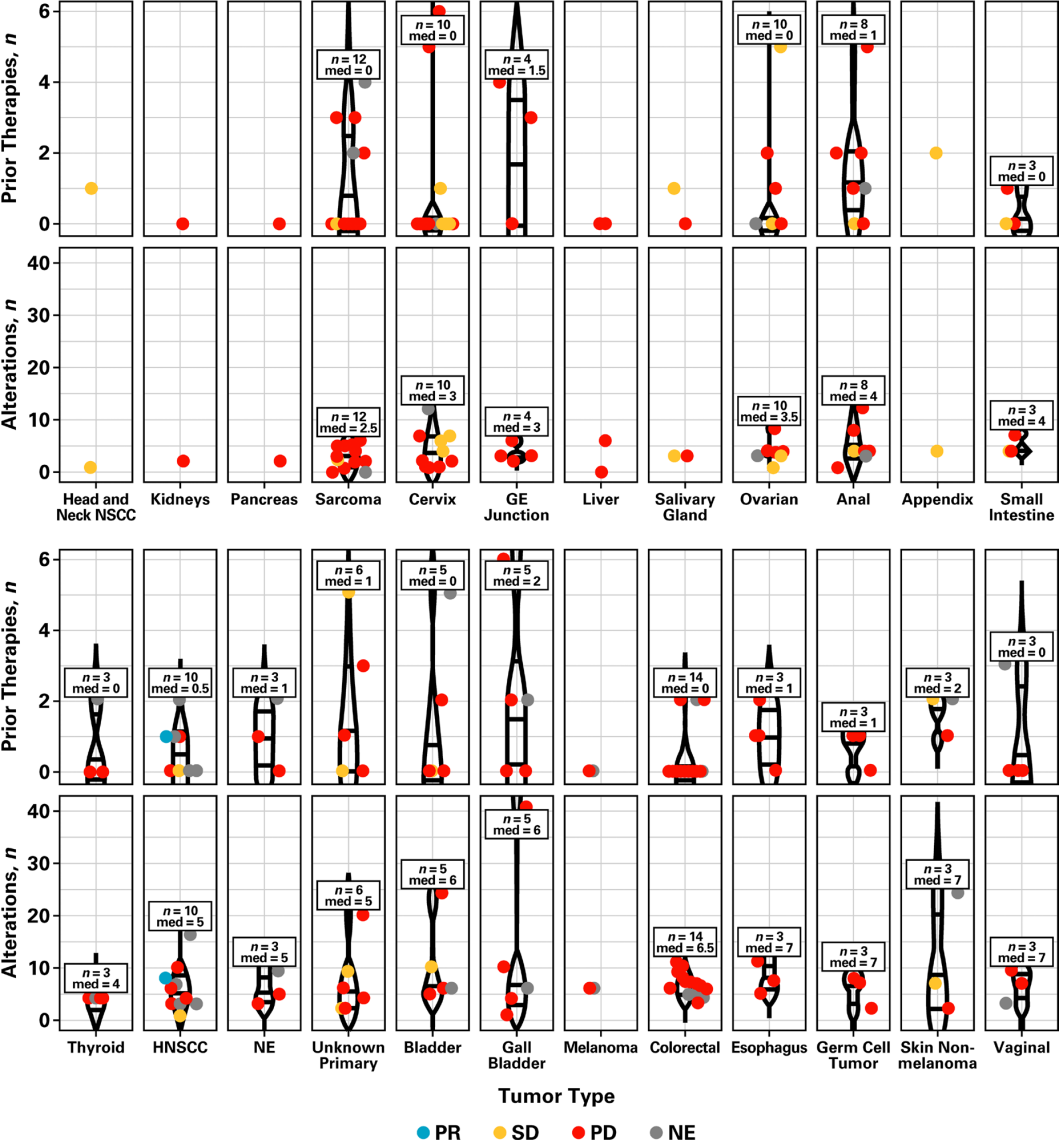
Number of prior therapies, genetic alterations, and tumor response by tumor type. Abbreviations: GE, gastroesophageal; HNSCC, head and neck squamous cell carcinoma; med, median; NE, neuroendocrine; NSCC, non-squamous cell carcinoma; PD, progressive disease; PR, partial response; SD, stable disease.

The association between response and the number of prior therapies or genetic alterations by tumor type was also assessed (Figure 2, Supplementary Figure 1, and Supplementary Table 1). Overall, tumors had a median of four mutations. Different tumor types showed different genetic alterations, which were not correlated with the number of previous lines of therapy patients had received (Pearson *r* = −0.037) or clinical benefit. Of note, patients who did not achieve clinical benefit at 16 weeks showed a higher frequency of mutations (range, 6 [4.1%] to 50 [34.2%]) in *APC*, *BRCA2*, *CDKN2A*, *EP300*, *KRAS*, *LRP1B*, *SMAD4*, and *TP53* (Supplementary Figure 2). Clinical benefit was observed primarily in patients with *PIK3CA* (14.8%; *n* = 8), *PTEN* (14.7%; *n* = 5), and *PIK3R1* mutations (14.3%; *n* = 1). Fewer genetic alterations were observed in patients with clinical benefit at 16 weeks (Supplementary Figure 2).

A total of 128 patients (87.7%) agreed to be followed for survival. The total survival follow-up was 2 years. Disease progression/relapse or death was reported in 76.7% of patients (112/146). The median time to progression was 1.9 months (95% confidence interval [CI], 1.8–2.3). The estimated probability of progression-free survival at 6 and 12 months was 14.1% (95% CI, 8.2–21.5) and 2.9% (95% CI, 0.6–8.6), respectively. Median overall survival was 6.3 months (95% CI, 5.2–8.8), with an estimated overall survival probability of 51.5% (95% CI, 43.0–59.4) at 6 months and 30.2% (95% CI, 22.6–38.1) at 12 months.

### Safety

All 146 patients had at least one adverse event regardless of relationship to the study drug. The most common adverse events reported in over 20% of patients were fatigue (49.3%), nausea (47.3%), decreased appetite (39.7%), diarrhea (34.9%), vomiting (29.5%), anxiety (28.1%), depression (28.1%), hyperglycemia (28.1%), increased aspartate aminotransferase (24.7%), and decreased weight (24.0%). Grade 3/4 adverse events were reported in 120 patients (82.2%). The most common grade 3/4 adverse events (>5% of patients) regardless of relationship to study drug were hyperglycemia (13.7%), fatigue (13.0%), increased aspartate aminotransferase (8.2%), increased alanine aminotransferase (8.2%), anxiety (7.5%), abdominal pain (6.2%), dyspnea (6.2%), depression (5.5%), and nausea (5.5%). Overall, 126 patients (86.3%) had at least one adverse event which was thought to be related to study drug (Table 3). The most common treatment-related adverse events were fatigue (34.2%), nausea (29.5%), hyperglycemia (24.0%), decreased appetite (23.3%), and diarrhea (22.6%). The most common adverse events (≥10 patients each) that led to study drug interruption or dose reduction included aspartate aminotransferase increase (13.7%), alanine aminotransferase increase (11.6%), hyperglycemia (9.6%), and fatigue (8.9%).

**Table 3:** Treatment-related adverse events occurring in >5% of patients

Adverse event, *n* (%)	Patients (*N* = 146)
Fatigue	50 (34.2)
Nausea	43 (29.5)
Hyperglycemia	35 (24.0)
Decreased appetite	34 (23.3)
Diarrhea	33 (22.6)
Aspartate aminotransferase increased	25 (17.1)
Vomiting	23 (15.8)
Depression	22 (15.1)
Alanine aminotransferase increased	21 (14.4)
Anxiety	20 (13.7)
Rash	20 (13.7)
Weight decreased	15 (10.3)
Dysgeusia	11 (7.5)
Dyspepsia	10 (6.8)
Mucosal inflammation	10 (6.8)
Insomnia	9 (6.2)

A total of 123 patients (84.2%) had at least one serious or significant adverse event; one serious adverse event (sepsis and pneumonia) led to death. Most patients had serious adverse events of grade 3 severity and only 14 patients (23.7%) had serious adverse events which were suspected to be related to study drug. Serious adverse events suspected to be related to study treatment were pneumonitis, esophageal perforation, seizure, vomiting, pancreatitis, mental status changes, erythema, palmar–plantar erythrodysesthesia syndrome, upper gastrointestinal hemorrhage, psychotic disorder, abdominal pain, nausea, anxiety, depression, failure to thrive, hyperglycemia, leukoencephalopathy, psoriasis, and rash. Observed laboratory abnormalities of interest included hyperglycemia (28.1%), aspartate aminotransferase increase (24.7%), and alanine aminotransferase increase (19.2%).

A total of 110 deaths occurred, 74.5% were more than 30 days after the last dose of treatment. Most deaths were due to disease progression (90.9%). Of the remaining deaths, seven patients died due to unknown reasons, one patient died from pneumonia, one patient died from exsanguination of the carotid artery, and one patient died due to adverse events (sepsis and pneumonia; both were considered to be unrelated to the study drug by the treating investigator).

## DISCUSSION

In this signal-seeking study, patients with PI3K pathway-activated tumors were matched to treatment with the highly selective pan-PI3K inhibitor, buparlisib. This study was part of the Novartis Signature Program, which investigated novel therapeutic strategies in a histology-agnostic, mutation-specific manner. The most common tumor types in this study were colorectal, sarcoma, and ovarian. Tumors had a median of four mutations; the lowest mutation burden was observed in sarcoma (median = 2.5) and the highest in esophagus, germ cell tumor, skin non-melanoma, and vaginal (median = 7). The number of prior therapies did not correlate with the number of genetic alterations observed. However, fewer genetic alterations were noted in patients with clinical benefit from buparlisib monotherapy.

The most common altered genes that were required at study entry were *PIK3CA* gene mutations or *PTEN* gene aberrations, with *PIK3CA* amplifications and *PIK3R1* gene mutations also identified. The most common altered co-occurring genes were *TP53*, *APC*, *KRAS*, *MLL2*, *CDKN2A*, *ERBB2*, *FBXW7*, *ARID1A*, and *RB1* (≥10 patients each) (Supplementary Table 1 and Supplementary Table 2). Mutations in *BRCA2*, *CDKN2A*, *EP300*, *KRAS*, *LRP1B*, *SMAD4*, *CCND*, *FGF3/4/19*, and *NFE2L2* were observed in patients with no clinical benefit. The patient who achieved a complete response had *GNAS* and *PIK3CA* mutations. Several patients achieved clinical benefit despite mutations in *ERBB2*, *ARID2*, *ATM*, *CCNE1*, *ARID1A*, *KRAS*, *TP53*, *SOX2*, *APC*, *FBXW7*, *RB1*, and *MLL2* (Supplementary Figure 2). However, the small number of patients in this study precludes correlation of mutation type with clinical outcome. Additionally, clinical benefit rate is often criticized as a reflection of true patient benefit, due to the inclusion of stable disease. In this study stable disease was required to last for at least 16 weeks; however, it is thought that a longer-term measure of stable disease for over 6 months is needed to imply anti-tumor effects [13].

Findings from this study suggest that efficacy of single-agent buparlisib in patients with advanced disease may be limited, despite patients being molecularly preselected. In the phase 2 BASALT-1 study (NCT01297491) clinical benefit was observed in 41% of patients with pretreated, metastatic non-small cell lung cancer, with an overall response rate of 3% [14]. Buparlisib also showed modest activity in relapsed or refractory non-Hodgkin lymphoma, with overall response rates of 11.5%, 22.7%, and 25.0% in patients with diffuse large B-cell lymphoma, mantle cell lymphoma, and follicular lymphoma, respectively (NCT01693614) [15]. Likewise, minimal efficacy in patients with glioblastoma has been observed (NCT01339052) [16].

Overall, buparlisib was well tolerated, with most patients not requiring dose interruptions or reductions, and showed clinical benefit for a subset of patients. Fatigue, nausea, hyperglycemia, decreased appetite, and diarrhea were the most commonly reported adverse events. Future efforts may provide additional insight into the efficacy of buparlisib in molecularly selected patient populations and may help determine potential for combination of buparlisib with other agents.

## MATERIALS AND METHODS

### Study design

This was a phase 2, open-label, multi-center, single-arm study in patients with solid or hematologic malignancies that were pre-identified to have activation of the PI3K pathway and whose disease had progressed on or after standard treatment. Patients received buparlisib 100 mg once daily until disease progression (as assessed by the investigator), unacceptable toxicity, death, or discontinuation from study treatment (e. g. withdrawal of consent, start of a new anti-cancer therapy, or investigator decision). Imaging assessments were performed every 8 weeks (± 4 days) after the first dose of study drug and every 16 weeks after the first 16 weeks on treatment.

The primary endpoint was the clinical benefit rate associated with buparlisib treatment based on local investigator assessment. Clinical benefit rate was defined as the proportion of patients with a best overall response of complete response, partial response, or stable disease at ≥16 weeks by RECIST 1.1 criteria or appropriate hematologic response criteria. The key secondary endpoint was to assess the overall response rate (overall response rate, i. e. complete or partial response) based on local investigator assessment. Other secondary endpoints were progression-free survival, duration of response, overall survival, and safety.

Patients were followed for safety analysis for 30 days after the last dose. All patients were followed for survival status every 3 months for 2 years after the last patient had enrolled in the study, regardless of treatment discontinuation reason (except if consent was withdrawn or patient was lost to follow-up), until November 12, 2015.

### Patients

Patients with a solid tumor or hematologic malignancy that had been pre-identified as PI3K pathway activated were eligible. Patients must have received at least one prior treatment for their recurrent, metastatic, and/or locally advanced disease with no remaining standard therapy options. Patients were excluded if they had received prior treatment with buparlisib. Patients with specific tumor types were not permitted if buparlisib had been previously shown to be ineffective as a single agent, had demonstrated early futility or success based on adaptive statistical design, or were currently being studied in other Novartis studies (e. g. endometrial cancer, glioblastoma and other central nervous system cancers, non-small cell lung cancer, prostate cancer, or breast cancer).

### Genomic profiling

Eligibility was based on gene aberration status as assessed by local testing in a clinical laboratory improvement amendments-certified laboratory prior to patient consent. PI3K pathway activation was defined as the presence of at least one of the following alterations: *PIK3CA*-activating gene mutation; *PTEN* loss-of-function gene mutation; phosphatase and tensin homolog (PTEN) protein loss as determined by immunohistochemistry (<10% of tumor cells expressing PTEN at 1+ level); *PIK3R1* loss-of-function gene mutations; and/or amplification of the *PIK3CA* gene. Genomic profiling was not part of the screening phase; following identification of a potential patient, investigators contacted Novartis for study enrollment consideration.

Patients were required to have archival tissue samples available for submission to allow for centralized molecular testing. If archival tissue was either not available or insufficient, collection of a fresh tumor biopsy was mandatory. Submission of biopsy samples was required prior to the first dose of buparlisib (unless otherwise agreed by Novartis and the investigator). Central testing for pathway activation was confirmed at Foundation Medicine [17] in a clinical laboratory improvement amendments-certified laboratory; next-generation sequencing was performed for a selection of more than 300 cancer-relevant genes and over 25 select introns associated with solid tumors. The comprehensive genetic profile offered a depth of coverage over 500× for the detection of low-frequency mutations. The assay provided a list of short variants (non-synonymous substitutions, indels, and frame shifts), copy number alterations (complete loss or amplifications), and rearrangements (genomic events altering order and continuity of genomic sequence) detected in a patient’s tumor sample. Germline mutations were filtered out, and the remaining genomic alterations were classified, based on state-of-the-art literature resources, into functionally known or likely, and functionally unknown aberrations [17, 18]. For these analyses, the mutational datasets for 100 archival and 15 fresh tumor biopsies were available. Analyses presented here are according to central laboratory assessment.

### Statistical analysis

Tumor cohorts were formed when at least four patients were enrolled with a particular tumor type. The total number of patients enrolled per tumor type was based on a patient-sparing, adaptive design, allowing early closure of non-responding groups and/or groups with early success.

Clinical benefit at 16 weeks was evaluated using a Bayesian adaptive statistical design that allowed dynamic borrowing of information across cohorts based on their degree of similarity in a hierarchical model, such that more borrowing occurred when the groups were consistent, and less borrowing occurred when the groups differed. Additionally, a clustering mechanism was incorporated that allowed borrowing within clusters but treated clusters separately. This design minimized the borrowing across groups that differed in clinical benefit rate and allowed for analyses to be conducted with small sample sizes for each cohort.

A minimum of 10 enrolled patients were required to evaluate early futility and at least 15 patients were required to evaluate early success for each tumor type. An early ‘go/no-go’ decision within each tumor cohort was made following these criteria. The enrollment of each group stopped early for futility if there was a less than 10% probability that the response rate in that group exceeded the historical rate of response. Alternatively, the enrollment of a group was stopped early for success if there was at least a 95% probability that the response rate in a group exceeded the historical rate of response. The final analysis occurred when both accrual and follow-up were complete for all groups. If there was at least a 90% probability that the response rate in a group exceeded the historical rate, the group was considered a success.

Clinical benefit rate, overall response rate, partial response, and complete response based on local investigator assessment were summarized, and 95% exact confidence intervals using the Clopper–Pearson method were reported. Correlation between specific genomic DNA profile based on central laboratory analysis and clinical benefit was explored. In addition, safety and tolerability of buparlisib was reported.

### Ethical oversight

This clinical study was designed, implemented, and reported in accordance with the Declaration of Helsinki and the International Conference on Harmonized Tripartite Guidelines for Good Clinical Practice, with applicable local regulations. The protocol and the proposed informed consent form were reviewed and approved by a central Institutional Review Board (Quorum) before study start. Informed consent has been obtained from all patients.

## SUPPLEMENTARY MATERIALS






