# Supplementary material for: Efficacy and safety of buparlisib, a PI3K inhibitor, in patients with malignancies harboring a PI3K pathway activation: a phase 2, open-label, single-arm study
Source: Oncotarget. 2019 Nov 5;10(60):6526–35. doi: 10.18632/oncotarget.27251 (PMC6849647; doi:10.18632/oncotarget.27251)
Supplement: Supplementary file 1 [file oncotarget-10-6526-s001.pdf]

Efficacy and safety of buparlisib, a PI3K inhibitor, in patients with malignancies harboring a PI3K pathway activation: a phase 2, open-label, single-arm study

SUPPLEMENTARY MATERIALS

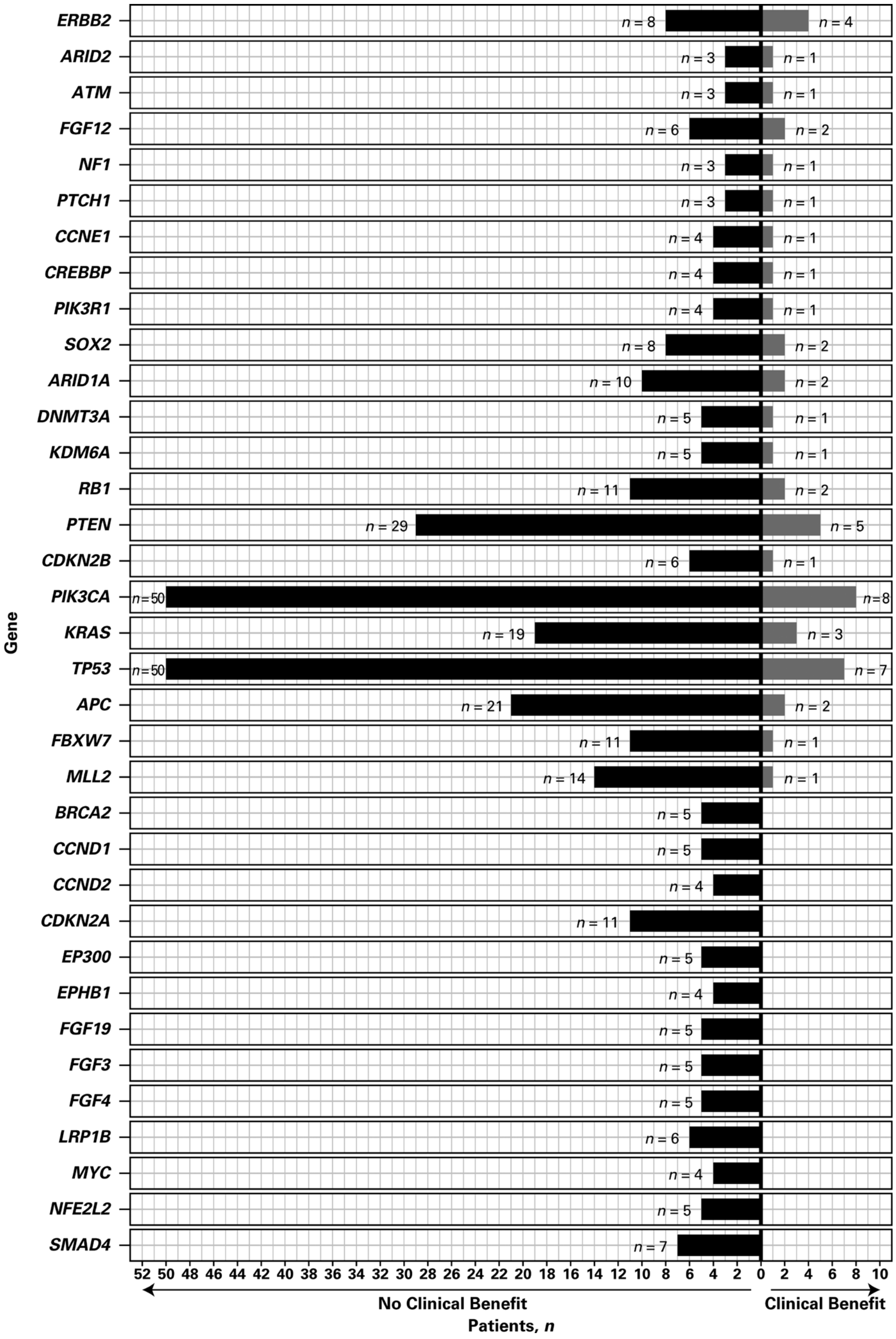

Supplementary Figure 1: Gene alterations in at least four patients by clinical benefit at week 16. Data indicate numbers of patients with each mutation (individual patients may have had more than one mutation).

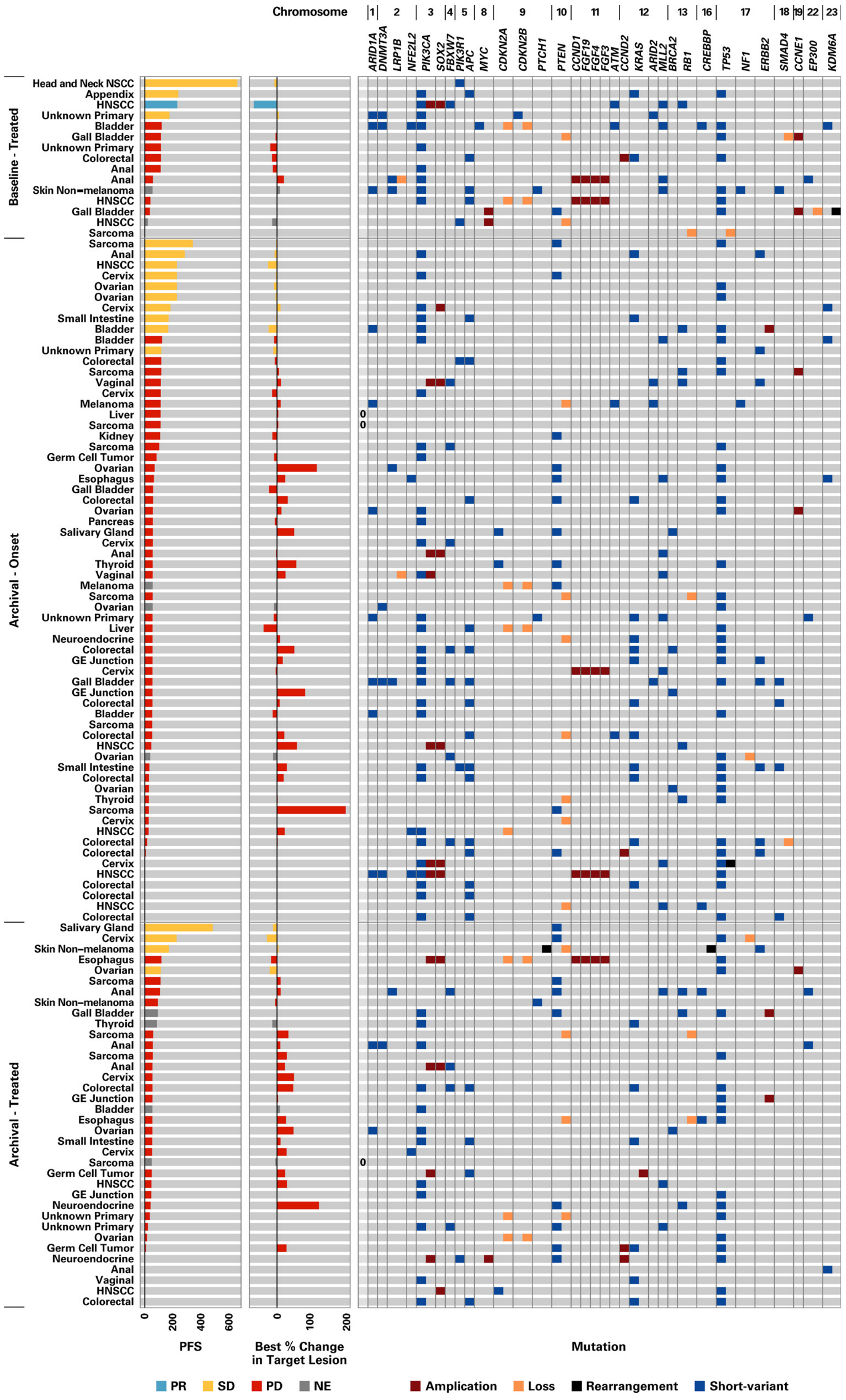

Supplementary Figure 2: Mutational landscape of patient biopsies, grouped based on time of collection with regard to trial start and prior therapies. Patients (rows) are sorted within each subgroup based on PFS, and bar charts are color coded based on BOR. Only functionally known and likely mutations are shown; different colored boxes indicate genera of mutations. Specific nucleotide substitutions of *PIK3CA*, *PIK3R1*, *KRAS*, and *RB1* are shown in Supplementary Table 2. Abbreviations: BOR, best overall response; GE, gastroesophageal; HNSCC, head and neck squamous cell carcinoma; NE, not evaluable; NSCC, non-squamous cell carcinoma; PD, progressive disease; PFS, progression-free survival; PR, partial response; SD, stable disease.

Supplementary Table 1: Gene mutation frequencies highlighting whether biopsy was collected without prior therapy (onset), after some prior therapy (treated), and overall. See Supplementary Table 1

Supplementary Table 2: Short-variant mutations in *PIK3CA*, *PIK3R1*, *KRAS*, and *RB1*. See Supplementary Table 2
