# Supplementary material for: Efficacy and safety of buparlisib, a PI3K inhibitor, in patients with malignancies harboring a PI3K pathway activation: a phase 2, open-label, single-arm study
Source: Oncotarget. 2019 Nov 5;10(60):6526–35. doi: 10.18632/oncotarget.27251 (PMC6849647; doi:10.18632/oncotarget.27251)
Supplement: Supplementary file 2 [file oncotarget-10-6526-s002.docx]

**Supplementary Table 1: Gene mutation frequencies highlighting whether biopsy was collected without prior therapy (onset), after some prior therapy (treated), and overall**

| **Gene** | **Onset** | **% Group (onset)** | **% Total (onset)** | **Treated** | **% Group (treated)** | **%  Total (treated)** | **Overall** | **% Total** |
| --- | --- | --- | --- | --- | --- | --- | --- | --- |
| *PIK3CA* | 32 | 50.0 | 27.8 | 25 | 49.0 | 21.7 | 57 | 49.6 |
| *TP53* | 32 | 50.0 | 27.8 | 25 | 49.0 | 21.7 | 57 | 49.6 |
| *PTEN* | 18 | 28.1 | 15.7 | 16 | 31.4 | 13.9 | 34 | 29.6 |
| *APC* | 15 | 23.4 | 13.0 | 8 | 15.7 | 7.0 | 23 | 20.0 |
| *KRAS* | 13 | 20.3 | 11.3 | 9 | 17.6 | 7.8 | 22 | 19.1 |
| *MLL2* | 8 | 12.5 | 7.0 | 7 | 13.7 | 6.1 | 15 | 13.0 |
| *RB1* | 6 | 9.4 | 5.2 | 7 | 13.7 | 6.1 | 13 | 11.3 |
| *ARID1A* | 7 | 10.9 | 6.1 | 5 | 9.8 | 4.3 | 12 | 10.4 |
| *ERBB2* | 9 | 14.1 | 7.8 | 3 | 5.9 | 2.6 | 12 | 10.4 |
| *FBXW7* | 7 | 10.9 | 6.1 | 5 | 9.8 | 4.3 | 12 | 10.4 |
| *CDKN2A* | 5 | 7.8 | 4.3 | 6 | 11.8 | 5.2 | 11 | 9.6 |
| *CDKN2B* | 2 | 3.1 | 1.7 | 5 | 9.8 | 4.3 | 7 | 6.1 |
| *SMAD4* | 5 | 7.8 | 4.3 | 2 | 3.9 | 1.7 | 7 | 6.1 |
| *DNMT3A* | 3 | 4.7 | 2.6 | 3 | 5.9 | 2.6 | 6 | 5.2 |
| *KDM6A* | 3 | 4.7 | 2.6 | 3 | 5.9 | 2.6 | 6 | 5.2 |
| *LRP1B* | 3 | 4.7 | 2.6 | 3 | 5.9 | 2.6 | 6 | 5.2 |
| *SOX2* | 3 | 4.7 | 2.6 | 3 | 5.9 | 2.6 | 6 | 5.2 |
| *BRCA2* | 4 | 6.2 | 3.5 | 1 | 2.0 | 0.9 | 5 | 4.3 |
| *CCND1* | 2 | 3.1 | 1.7 | 3 | 5.9 | 2.6 | 5 | 4.3 |
| *CCNE1* | 2 | 3.1 | 1.7 | 3 | 5.9 | 2.6 | 5 | 4.3 |
| *CREBBP* | 1 | 1.6 | 0.9 | 4 | 7.8 | 3.5 | 5 | 4.3 |
| *EP300* | 1 | 1.6 | 0.9 | 4 | 7.8 | 3.5 | 5 | 4.3 |
| *FGF19* | 2 | 3.1 | 1.7 | 3 | 5.9 | 2.6 | 5 | 4.3 |
| *FGF3* | 2 | 3.1 | 1.7 | 3 | 5.9 | 2.6 | 5 | 4.3 |
| *FGF4* | 2 | 3.1 | 1.7 | 3 | 5.9 | 2.6 | 5 | 4.3 |
| *NFE2L2* | 3 | 4.7 | 2.6 | 2 | 3.9 | 1.7 | 5 | 4.3 |
| *PIK3R1* | 2 | 3.1 | 1.7 | 3 | 5.9 | 2.6 | 5 | 4.3 |
| *ARID2* | 3 | 4.7 | 2.6 | 1 | 2.0 | 0.9 | 4 | 3.5 |
| *ATM* | 2 | 3.1 | 1.7 | 2 | 3.9 | 1.7 | 4 | 3.5 |
| *CCND2* | 1 | 1.6 | 0.9 | 3 | 5.9 | 2.6 | 4 | 3.5 |
| *MYC* | 0 | 0.0 | 0.0 | 4 | 7.8 | 3.5 | 4 | 3.5 |
| *NF1* | 2 | 3.1 | 1.7 | 2 | 3.9 | 1.7 | 4 | 3.5 |
| *PTCH1* | 1 | 1.6 | 0.9 | 3 | 5.9 | 2.6 | 4 | 3.5 |
| *AKT2* | 2 | 3.1 | 1.7 | 1 | 2.0 | 0.9 | 3 | 2.6 |
| *BCOR* | 1 | 1.6 | 0.9 | 2 | 3.9 | 1.7 | 3 | 2.6 |
| *CDK12* | 3 | 4.7 | 2.6 | 0 | 0.0 | 0.0 | 3 | 2.6 |
| *CIC* | 1 | 1.6 | 0.9 | 2 | 3.9 | 1.7 | 3 | 2.6 |
| *ERBB4* | 3 | 4.7 | 2.6 | 0 | 0.0 | 0.0 | 3 | 2.6 |
| *FGF12* | 1 | 1.6 | 0.9 | 2 | 3.9 | 1.7 | 3 | 2.6 |
| *FGF23* | 1 | 1.6 | 0.9 | 2 | 3.9 | 1.7 | 3 | 2.6 |
| *FGF6* | 1 | 1.6 | 0.9 | 2 | 3.9 | 1.7 | 3 | 2.6 |
| *FGFR3* | 2 | 3.1 | 1.7 | 1 | 2.0 | 0.9 | 3 | 2.6 |
| *KDM5A* | 1 | 1.6 | 0.9 | 2 | 3.9 | 1.7 | 3 | 2.6 |
| *PBRM1* | 1 | 1.6 | 0.9 | 2 | 3.9 | 1.7 | 3 | 2.6 |
| *AKT1* | 0 | 0.0 | 0.0 | 2 | 3.9 | 1.7 | 2 | 1.7 |
| *ARFRP1* | 2 | 3.1 | 1.7 | 0 | 0.0 | 0.0 | 2 | 1.7 |
| *BACH1* | 0 | 0.0 | 0.0 | 2 | 3.9 | 1.7 | 2 | 1.7 |
| *BARD1* | 2 | 3.1 | 1.7 | 0 | 0.0 | 0.0 | 2 | 1.7 |
| *BCORL1* | 2 | 3.1 | 1.7 | 0 | 0.0 | 0.0 | 2 | 1.7 |
| *BRAF* | 2 | 3.1 | 1.7 | 0 | 0.0 | 0.0 | 2 | 1.7 |
| *BRCA1* | 1 | 1.6 | 0.9 | 1 | 2.0 | 0.9 | 2 | 1.7 |
| *BRIP1* | 1 | 1.6 | 0.9 | 1 | 2.0 | 0.9 | 2 | 1.7 |
| *CARD11* | 1 | 1.6 | 0.9 | 1 | 2.0 | 0.9 | 2 | 1.7 |
| *CASP8* | 1 | 1.6 | 0.9 | 1 | 2.0 | 0.9 | 2 | 1.7 |
| *CDH1* | 0 | 0.0 | 0.0 | 2 | 3.9 | 1.7 | 2 | 1.7 |
| *CDKN1B* | 1 | 1.6 | 0.9 | 1 | 2.0 | 0.9 | 2 | 1.7 |
| *CTCF* | 2 | 3.1 | 1.7 | 0 | 0.0 | 0.0 | 2 | 1.7 |
| *CTNNA1* | 2 | 3.1 | 1.7 | 0 | 0.0 | 0.0 | 2 | 1.7 |
| *CTNNB1* | 1 | 1.6 | 0.9 | 1 | 2.0 | 0.9 | 2 | 1.7 |
| *DAXX* | 2 | 3.1 | 1.7 | 0 | 0.0 | 0.0 | 2 | 1.7 |
| *EPHA5* | 2 | 3.1 | 1.7 | 0 | 0.0 | 0.0 | 2 | 1.7 |
| *EPHB1* | 2 | 3.1 | 1.7 | 0 | 0.0 | 0.0 | 2 | 1.7 |
| *ERBB3* | 1 | 1.6 | 0.9 | 1 | 2.0 | 0.9 | 2 | 1.7 |
| *FAM123B* | 2 | 3.1 | 1.7 | 0 | 0.0 | 0.0 | 2 | 1.7 |
| *FGFR1* | 1 | 1.6 | 0.9 | 1 | 2.0 | 0.9 | 2 | 1.7 |
| *FGFR2* | 0 | 0.0 | 0.0 | 2 | 3.9 | 1.7 | 2 | 1.7 |
| *FLT1* | 1 | 1.6 | 0.9 | 1 | 2.0 | 0.9 | 2 | 1.7 |
| *GATA3* | 1 | 1.6 | 0.9 | 1 | 2.0 | 0.9 | 2 | 1.7 |
| *GNAS* | 1 | 1.6 | 0.9 | 1 | 2.0 | 0.9 | 2 | 1.7 |
| *GRIN2A* | 2 | 3.1 | 1.7 | 0 | 0.0 | 0.0 | 2 | 1.7 |
| *IKZF1* | 2 | 3.1 | 1.7 | 0 | 0.0 | 0.0 | 2 | 1.7 |
| *JAK2* | 2 | 3.1 | 1.7 | 0 | 0.0 | 0.0 | 2 | 1.7 |
| *MAP2K1* | 1 | 1.6 | 0.9 | 1 | 2.0 | 0.9 | 2 | 1.7 |
| *MAP3K1* | 1 | 1.6 | 0.9 | 1 | 2.0 | 0.9 | 2 | 1.7 |
| *MAP3K13* | 0 | 0.0 | 0.0 | 2 | 3.9 | 1.7 | 2 | 1.7 |
| *MDM2* | 1 | 1.6 | 0.9 | 1 | 2.0 | 0.9 | 2 | 1.7 |
| *MSH2* | 2 | 3.1 | 1.7 | 0 | 0.0 | 0.0 | 2 | 1.7 |
| *MTOR* | 0 | 0.0 | 0.0 | 2 | 3.9 | 1.7 | 2 | 1.7 |
| *MUTYH* | 1 | 1.6 | 0.9 | 1 | 2.0 | 0.9 | 2 | 1.7 |
| *MYST3* | 1 | 1.6 | 0.9 | 1 | 2.0 | 0.9 | 2 | 1.7 |
| *NF2* | 1 | 1.6 | 0.9 | 1 | 2.0 | 0.9 | 2 | 1.7 |
| *NOTCH1* | 0 | 0.0 | 0.0 | 2 | 3.9 | 1.7 | 2 | 1.7 |
| *NOTCH4* | 2 | 3.1 | 1.7 | 0 | 0.0 | 0.0 | 2 | 1.7 |
| *NRAS* | 1 | 1.6 | 0.9 | 1 | 2.0 | 0.9 | 2 | 1.7 |
| *NUP93* | 1 | 1.6 | 0.9 | 1 | 2.0 | 0.9 | 2 | 1.7 |
| *PIK3CG* | 1 | 1.6 | 0.9 | 1 | 2.0 | 0.9 | 2 | 1.7 |
| *RAF1* | 2 | 3.1 | 1.7 | 0 | 0.0 | 0.0 | 2 | 1.7 |
| *RNF43* | 2 | 3.1 | 1.7 | 0 | 0.0 | 0.0 | 2 | 1.7 |
| *SETD2* | 1 | 1.6 | 0.9 | 1 | 2.0 | 0.9 | 2 | 1.7 |
| *SMARCB1* | 1 | 1.6 | 0.9 | 1 | 2.0 | 0.9 | 2 | 1.7 |
| *SPEN* | 1 | 1.6 | 0.9 | 1 | 2.0 | 0.9 | 2 | 1.7 |
| *STK11* | 1 | 1.6 | 0.9 | 1 | 2.0 | 0.9 | 2 | 1.7 |
| *TERT* | 1 | 1.6 | 0.9 | 1 | 2.0 | 0.9 | 2 | 1.7 |
| *XRCC3* | 2 | 3.1 | 1.7 | 0 | 0.0 | 0.0 | 2 | 1.7 |
| *ZNF217* | 1 | 1.6 | 0.9 | 1 | 2.0 | 0.9 | 2 | 1.7 |
| *ABL1* | 1 | 1.6 | 0.9 | 0 | 0.0 | 0.0 | 1 | 0.9 |
| *ALK* | 1 | 1.6 | 0.9 | 0 | 0.0 | 0.0 | 1 | 0.9 |
| *AMER1* | 1 | 1.6 | 0.9 | 0 | 0.0 | 0.0 | 1 | 0.9 |
| *APCDD1* | 1 | 1.6 | 0.9 | 0 | 0.0 | 0.0 | 1 | 0.9 |
| *AR* | 1 | 1.6 | 0.9 | 0 | 0.0 | 0.0 | 1 | 0.9 |
| *ARNT2* | 0 | 0.0 | 0.0 | 1 | 2.0 | 0.9 | 1 | 0.9 |
| *ASXL1* | 1 | 1.6 | 0.9 | 0 | 0.0 | 0.0 | 1 | 0.9 |
| *ATR* | 1 | 1.6 | 0.9 | 0 | 0.0 | 0.0 | 1 | 0.9 |
| *ATRX* | 0 | 0.0 | 0.0 | 1 | 2.0 | 0.9 | 1 | 0.9 |
| *AXIN1* | 0 | 0.0 | 0.0 | 1 | 2.0 | 0.9 | 1 | 0.9 |
| *AXL* | 0 | 0.0 | 0.0 | 1 | 2.0 | 0.9 | 1 | 0.9 |
| *BAP1* | 1 | 1.6 | 0.9 | 0 | 0.0 | 0.0 | 1 | 0.9 |
| *BCL2L1* | 0 | 0.0 | 0.0 | 1 | 2.0 | 0.9 | 1 | 0.9 |
| *CCND3* | 1 | 1.6 | 0.9 | 0 | 0.0 | 0.0 | 1 | 0.9 |
| *CDC73* | 1 | 1.6 | 0.9 | 0 | 0.0 | 0.0 | 1 | 0.9 |
| *CDK4* | 1 | 1.6 | 0.9 | 0 | 0.0 | 0.0 | 1 | 0.9 |
| *CDK6* | 0 | 0.0 | 0.0 | 1 | 2.0 | 0.9 | 1 | 0.9 |
| *CHD4* | 0 | 0.0 | 0.0 | 1 | 2.0 | 0.9 | 1 | 0.9 |
| *CHEK2* | 1 | 1.6 | 0.9 | 0 | 0.0 | 0.0 | 1 | 0.9 |
| *CHUK* | 0 | 0.0 | 0.0 | 1 | 2.0 | 0.9 | 1 | 0.9 |
| *CRKL* | 0 | 0.0 | 0.0 | 1 | 2.0 | 0.9 | 1 | 0.9 |
| *CSF1R* | 1 | 1.6 | 0.9 | 0 | 0.0 | 0.0 | 1 | 0.9 |
| *DNAH2* | 1 | 1.6 | 0.9 | 0 | 0.0 | 0.0 | 1 | 0.9 |
| *EGFR* | 1 | 1.6 | 0.9 | 0 | 0.0 | 0.0 | 1 | 0.9 |
| *EMSY* | 1 | 1.6 | 0.9 | 0 | 0.0 | 0.0 | 1 | 0.9 |
| *ESR1* | 0 | 0.0 | 0.0 | 1 | 2.0 | 0.9 | 1 | 0.9 |
| *EZH2* | 1 | 1.6 | 0.9 | 0 | 0.0 | 0.0 | 1 | 0.9 |
| *FANCA* | 0 | 0.0 | 0.0 | 1 | 2.0 | 0.9 | 1 | 0.9 |
| *FAS* | 0 | 0.0 | 0.0 | 1 | 2.0 | 0.9 | 1 | 0.9 |
| *FAT1* | 0 | 0.0 | 0.0 | 1 | 2.0 | 0.9 | 1 | 0.9 |
| *FAT3* | 1 | 1.6 | 0.9 | 0 | 0.0 | 0.0 | 1 | 0.9 |
| *FHAD1* | 0 | 0.0 | 0.0 | 1 | 2.0 | 0.9 | 1 | 0.9 |
| *FLT3* | 1 | 1.6 | 0.9 | 0 | 0.0 | 0.0 | 1 | 0.9 |
| *HRAS* | 1 | 1.6 | 0.9 | 0 | 0.0 | 0.0 | 1 | 0.9 |
| *IGF1R* | 0 | 0.0 | 0.0 | 1 | 2.0 | 0.9 | 1 | 0.9 |
| *INHBA* | 1 | 1.6 | 0.9 | 0 | 0.0 | 0.0 | 1 | 0.9 |
| *IRS2* | 1 | 1.6 | 0.9 | 0 | 0.0 | 0.0 | 1 | 0.9 |
| *MECOM* | 1 | 1.6 | 0.9 | 0 | 0.0 | 0.0 | 1 | 0.9 |
| *MED12* | 0 | 0.0 | 0.0 | 1 | 2.0 | 0.9 | 1 | 0.9 |
| *MEN1* | 1 | 1.6 | 0.9 | 0 | 0.0 | 0.0 | 1 | 0.9 |
| *MET* | 0 | 0.0 | 0.0 | 1 | 2.0 | 0.9 | 1 | 0.9 |
| *MITF* | 1 | 1.6 | 0.9 | 0 | 0.0 | 0.0 | 1 | 0.9 |
| *NCOR1* | 0 | 0.0 | 0.0 | 1 | 2.0 | 0.9 | 1 | 0.9 |
| *NOTCH2* | 0 | 0.0 | 0.0 | 1 | 2.0 | 0.9 | 1 | 0.9 |
| *NOTCH3* | 0 | 0.0 | 0.0 | 1 | 2.0 | 0.9 | 1 | 0.9 |
| *PALB2* | 1 | 1.6 | 0.9 | 0 | 0.0 | 0.0 | 1 | 0.9 |
| *PARK2* | 0 | 0.0 | 0.0 | 1 | 2.0 | 0.9 | 1 | 0.9 |
| *PIK3C2G* | 1 | 1.6 | 0.9 | 0 | 0.0 | 0.0 | 1 | 0.9 |
| *PIK3R2* | 0 | 0.0 | 0.0 | 1 | 2.0 | 0.9 | 1 | 0.9 |
| *PPP2R1A* | 1 | 1.6 | 0.9 | 0 | 0.0 | 0.0 | 1 | 0.9 |
| *PRDM1* | 0 | 0.0 | 0.0 | 1 | 2.0 | 0.9 | 1 | 0.9 |
| *PRKAR1A* | 0 | 0.0 | 0.0 | 1 | 2.0 | 0.9 | 1 | 0.9 |
| *PRSS8* | 1 | 1.6 | 0.9 | 0 | 0.0 | 0.0 | 1 | 0.9 |
| *PTPN11* | 0 | 0.0 | 0.0 | 1 | 2.0 | 0.9 | 1 | 0.9 |
| *RAD50* | 1 | 1.6 | 0.9 | 0 | 0.0 | 0.0 | 1 | 0.9 |
| *RNF11* | 1 | 1.6 | 0.9 | 0 | 0.0 | 0.0 | 1 | 0.9 |
| *RPTOR* | 0 | 0.0 | 0.0 | 1 | 2.0 | 0.9 | 1 | 0.9 |
| *RUNX1* | 0 | 0.0 | 0.0 | 1 | 2.0 | 0.9 | 1 | 0.9 |
| *SF3B1* | 0 | 0.0 | 0.0 | 1 | 2.0 | 0.9 | 1 | 0.9 |
| *SMAD2* | 1 | 1.6 | 0.9 | 0 | 0.0 | 0.0 | 1 | 0.9 |
| *SMARCA4* | 1 | 1.6 | 0.9 | 0 | 0.0 | 0.0 | 1 | 0.9 |
| *SPTA1* | 0 | 0.0 | 0.0 | 1 | 2.0 | 0.9 | 1 | 0.9 |
| *STAT4* | 1 | 1.6 | 0.9 | 0 | 0.0 | 0.0 | 1 | 0.9 |
| *TET2* | 1 | 1.6 | 0.9 | 0 | 0.0 | 0.0 | 1 | 0.9 |
| *TOP1* | 0 | 0.0 | 0.0 | 1 | 2.0 | 0.9 | 1 | 0.9 |
| *TRRAP* | 1 | 1.6 | 0.9 | 0 | 0.0 | 0.0 | 1 | 0.9 |
| *TSC1* | 0 | 0.0 | 0.0 | 1 | 2.0 | 0.9 | 1 | 0.9 |
| *TSC2* | 1 | 1.6 | 0.9 | 0 | 0.0 | 0.0 | 1 | 0.9 |
| *TTK* | 0 | 0.0 | 0.0 | 1 | 2.0 | 0.9 | 1 | 0.9 |
| *ZNF703* | 1 | 1.6 | 0.9 | 0 | 0.0 | 0.0 | 1 | 0.9 |

The “% Group” columns detail the number of patients in the group with the mutated gene present in the tumor biopsy. The “% Total” columns detail the number of patients out of the entire study that had a biopsy in the group with a mutation present in that gene. Overall and % total columns illustrate overall mutation detection regardless of when the biopsy was collected.
