# Supplementary material for: Efficacy and safety of buparlisib, a PI3K inhibitor, in patients with malignancies harboring a PI3K pathway activation: a phase 2, open-label, single-arm study
Source: Oncotarget. 2019 Nov 5;10(60):6526–35. doi: 10.18632/oncotarget.27251 (PMC6849647; doi:10.18632/oncotarget.27251)
Supplement: Supplementary file 3 [file oncotarget-10-6526-s003.docx]

**Supplementary Table 2: Short-variant mutations in *PIK3CA*, *PIK3R1*, *KRAS*, and *RB1***

| **Tumor** **type** | **Short-variant mutations** | | | |
| --- | --- | --- | --- | --- |
|  | ***PIK3CA*** | ***PIK3R1*** | ***KRAS*** | ***RB1*** |
| Head and neck NSCC | - | F456_Q457>I.FQ | - | - |
| Appendix | E542K | - | G12C | - |
| HNSCC | E545K | - | - | R251* |
| Unknown primary | E545K | - | - | - |
| Bladder | E418K E970K | - | - | - |
| Gall bladder | - | - | - | - |
| Unknown primary | E418K E542K | - | - | - |
| Colorectal | - | - | G12R | - |
| Anal | E545K | - | - | - |
| Anal | E545K | - | - | - |
| Skin non-melanoma | H1047L | - | - | - |
| HNSCC | H1047R | - | - | - |
| Gall bladder | - | - | - | - |
| HNSCC | - | K567_L570del | - | - |
| Sarcoma | - | - | - | - |
| Sarcoma | - | - | - | - |
| Anal | E542K | - | G12V | - |
| HNSCC | - | - | - | - |
| Cervix | E545K | - | - | - |
| Ovarian | - | - | - | - |
| Ovarian | - | - | - | - |
| Cervix | H1047R | - | - | - |
| Small intestine | M1043I | - | G12D | - |
| Bladder | E545K E970K | - | - | Q217* |
| Bladder | E545K | - | - | - |
| Unknown primary | - | - | - | - |
| Colorectal | - | R534* | - | - |
| Sarcoma | - | - | - | Splice |
| Vaginal | - | - | - | E539* |
| Cervix | E545K | - | - | - |
| Melanoma | - | - | - | - |
| Liver | - | - | - | - |
| Sarcoma | - | - | - | - |
| Kidney | - | - | - | - |
| Sarcoma | G1049R | - | - | - |
| Germ cell tumor | E545K | - | - | - |
| Ovarian | - | - | - | - |
| Esophagus | - | - | - | - |
| Gall bladder | - | - | - | - |
| Colorectal | - | - | G12D | - |
| Ovarian | T1025N | - | - | - |
| Pancreas | H1047Y | - | - | - |
| Salivary gland | - | - | - | - |
| Cervix | E542K | - | - | - |
| Anal | - | - | - | - |
| Thyroid | - | - | - | - |
| Vaginal | E545K | - | - | - |
| Melanoma | - | - | - | - |
| Sarcoma | - | - | - | - |
| Ovarian | - | - | - | - |
| Unknown primary | E545G | - | G12V | - |
| Liver | V105_E109del | - | - | - |
| Neuroendocrine | - | - | G13D | - |
| Colorectal | N345K | - | A146T | - |
| GE junction | R88Q | - | G12D | - |
| Cervix | E542K | - | - | - |
| Gall bladder | R93Q | - | - | - |
| GE junction | - | - | - | - |
| Colorectal | M1043I | - | G12C | - |
| Bladder | E542K E726K | - | - | - |
| Sarcoma | - | - | - | - |
| Colorectal | - | - | A146T | - |
| HNSCC | - | - | - | Y813fs*10 |
| Ovarian | - | - | - | - |
| Small intestine | E545K | V662fs*25 | A146P | - |
| Colorectal | Q546K | - | A59E | - |
| Ovarian | - | - | - | - |
| Thyroid | - | - | - | D68fs*37 |
| Sarcoma | - | - | - | - |
| Cervix | - | - | - | - |
| HNSCC | G118D | - | - | - |
| Colorectal | E542K | - | A11_G12insG | - |
| Colorectal | - | - | - | - |
| Cervix | E545K E726K | - | - | - |
| HNSCC | E542K | - | - | - |
| Colorectal | E545K | - | G12D | - |
| Colorectal | E542K | - | - | - |
| HNSCC | - | - | - | - |
| Colorectal | Q546R | - | - | - |
| Salivary gland | - | - | - | - |
| Cervix | - | - | - | - |
| Skin non-melanoma | - | - | - | - |
| Esophagus | - | - | - | - |
| Ovarian | - | - | - | - |
| Sarcoma | - | - | - | - |
| Anal | - | - | - | S149* R579* |
| Skin non-melanoma | - | - | - | - |
| Gall bladder | E365K | - | - | Q471* |
| Thyroid | Q546K | - | G12C | - |
| Sarcoma | - | - | - | - |
| Anal | E453K E545K | - | - | - |
| Sarcoma | - | - | - | - |
| Anal | - | - | - | - |
| Cervix | - | - | - | - |
| Colorectal | E542K | - | A146T | - |
| GE junction | - | - | - | - |
| Bladder | N1044K | - | - | - |
| Esophagus | - | - | - | - |
| Ovarian | H1047R | - | - | - |
| Small intestine | E545K | - | G12V | - |
| Cervix | - | - | - | - |
| Sarcoma | - | - | - | - |
| Germ cell tumor | - | - | - | - |
| HNSCC | E542K | - | - | - |
| GE junction | N345K | - | - | - |
| Neuroendocrine | - | - | - | Splice site 1499─1G>T |
| Unknown primary | - | - | - | - |
| Unknown primary | E542K | - | - | - |
| Ovarian | - | - | - | - |
| Germ cell tumor | - | - | A146P | - |
| Neuroendocrine | - | R649Q | - | - |
| Anal | - | - | - | - |
| Vaginal | H1047R | - | Q61H | - |
| HNSCC | - | - | - | - |
| Colorectal | E542K | - | G13D | - |

Abbreviations: del, deletion; GE, gastroesophageal; HNSCC, head and neck squamous cell carcinoma; ins, insertion; NSCC, non-squamous cell carcinoma.
